# The CpG-sites of the CBX3 ubiquitous chromatin opening element are critical structural determinants for the anti-silencing function

**DOI:** 10.1038/s41598-017-04212-8

**Published:** 2017-08-11

**Authors:** Jessica Kunkiel, Natascha Gödecke, Mania Ackermann, Dirk Hoffmann, Axel Schambach, Nico Lachmann, Dagmar Wirth, Thomas Moritz

**Affiliations:** 1 Reprogramming and Gene Therapy Group, REBIRTH Cluster of Excellence, Hannover Medical School, 30625 Hannover, Germany; 2 Institute of Experimental Hematology, Hannover Medical School, 30625 Hannover, Germany; 3 Model Systems for Infection and Immunity Group, Helmholtz Centre for Infection Research, 38124 Braunschweig, Germany; 4 Translational Hematology of Congenital Diseases Junior-Group, REBIRTH Cluster of Excellence, Hannover Medical School, 30625 Hannover, Germany; 5 Division of Hematology/Oncology, Boston Children’s Hospital, Boston, MA USA

## Abstract

Suppression of therapeutic transgene expression from retroviral gene therapy vectors by epigenetic defence mechanisms represents a problem that is particularly encountered in pluripotent stem cells (PSCs) and their differentiated progeny. Transgene expression in these cells, however, can be stabilised by CpG-rich ubiquitous chromatin opening elements (UCOEs). In this context we recently demonstrated profound anti-silencing properties for the small (679 bp) CBX3-UCO element and we now confirmed this observation in the context of the defined murine chromosomal loci ROSA26 and TIGRE. Moreover, since the structural basis for the anti-silencing activity of UCOEs has remained poorly defined, we interrogated various CBX3 subfragments in the context of lentiviral vectors and murine PSCs. We demonstrated marked though distinct anti-silencing activity in the pluripotent state and during PSC-differentiation for several of the CBX3 subfragments. This activity was significantly correlated with CpG content as well as endogenous transcriptional activity. Interestingly, also a scrambled CBX3 version with preserved CpG-sites retained the anti-silencing activity despite the lack of endogenous promoter activity. Our data therefore highlight the importance of CpG-sites and transcriptional activity for UCOE functionality and suggest contributions from different mechanisms to the overall anti-silencing function of the CBX3 element.

## Introduction

Although the design of lentiviral vectors has improved substantially over the last decade, even modern SIN-lentiviral vectors remain subject to positional effects that can lead to silenced or variegated transgene expression^1, 2^. In particular pluripotent stem cells are problematic in this respect, as these cells harbour strong intrinsic defence mechanisms against foreign and especially viral DNA^3–7^. Moreover, the differentiation of pluripotent stem cells towards more mature cell types involves genome wide epigenetic remodelling with a potentially detrimental impact on transgene expression. To evade this transcriptional repression so called ubiquitous chromatin opening elements (UCOEs) have been applied^8^. In previous studies, we and others have demonstrated the ability of UCOEs to establish stable expression of lentiviral transgenes driven by both viral and physiological promoters in murine and human pluripotent as well as multipotent target cells and thereof differentiated progeny^9–13^, thus demonstrating broad applicability of UCOEs.

The most commonly used UCOE is derived from the human *HNRPA2B1-CBX3* housekeeping gene locus. This element contains two divergently transcribed promoters, the HNRPA2B1 promoter and the CBX3 promoter which are both spanned by a large stretch of non-methylated CpG-islands (CGI)^11, 13^. A central 1.5 kb sequence containing the anti-silencing activity (A2UCOE) has been identified, which is associated with a permissive chromatin structure marked by hyperacetylation of histones H3 and H4, trimethylation of lysine 4 on histone H3 (H3K4me3), and the lack of H1^14^. We have recently shown that a much smaller sequence comprising only the 0.7 kb CBX3 moiety of the A2UCOE (referred to as CBX3) can effectively protect SIN-lentiviral transgene expression in murine and human pluripotent cells as well as their hematopoietic derivatives^15^. The CBX3 prevented CpG methylation of a spleen focus forming virus (SFFV) promoter and reduced the presence of repressive chromatin marks (e.g. H3K9me3 and H3K27me3) while introducing active marks such as H3K4me3 and PhosPol2.

While mechanistically the anti-silencing function of the UCOEs has been attributed to the presence of non-methylated CGIs, a feature shared by all known UCOEs, up to now the anti-silencing properties of the UCOE have not been linked to specific structural features. To potentially identify such features within the CBX3 structure we have generated a number of CBX3-derived subfragments and analysed the functionality of these subfragments in the context of lentiviral vectors and a broad range of integration sites within murine ESCs and iPSCs as well as their differentiated progeny. In addition, we have investigated the relevance of the CpG-islands for the anti-silencing properties by employing two synthetic scrambled-CBX3 versions with either maintained or destroyed CpG-sites. Moreover, to explore the relevance of transcriptional activity for the anti-silencing function we examined the promoter activity of our CBX3 subfragments. So far all investigations assessing UCOE function have been performed in bulk cell populations with varying integration sites while a direct comparison of expression cassettes with and without UCOE in a defined integration site and chromosomal architecture has not yet been performed. Accordingly, it is not known if UCOEs can protect from silencing irrespective of the nature of the chromosomal integration site. Therefore, we now have examined the anti-silencing ability of the CBX3 at two defined chromosomal loci, the ROSA26^16^ and the TIGRE locus T1^17^, in single-cell derived mESC clones utilising recombinase mediated cassette exchange (RMCE).

## Results

### CBX3 subfragments and their activity in pluripotent stem cells

**Figure 1:**
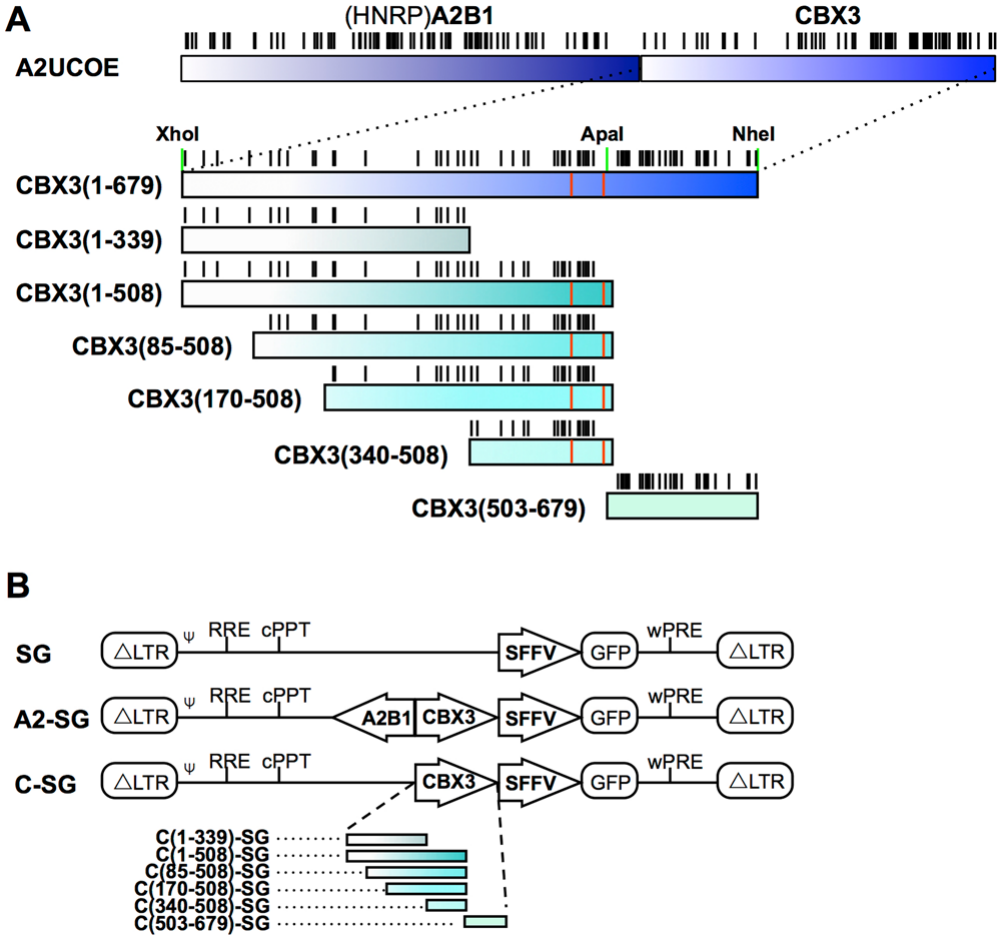
CBX3 subfragments and lentiviral vectors. (**A**) Schematic representation of the CBX3 element with its CpG-sites (black bars) and deleted splice sites (red bars) as well as the CBX3 subfragments obtained by PCR (CBX3(1-339) - CBX3(340-508)) and the CBX3(503-679) subfragment generated by restriction digestion with XhoI and ApaI. (**B**) Third-generation self-inactivating (SIN) lentiviral constructs expressing an eGFP cDNA from the spleen focus forming virus (SFFV) promoter in the absence or presence of either the 1.5 kb A2UCOE, the 679 bp CBX3 or any of the CBX3 subfragments. Abbreviations: ΔLTR: Long terminal repeat harboring the SIN mutation in the U3 region of the LTR; ψ: extended encapsidation signal; RRE: Rev-response element; cPPT: central polypurine tract; GFP: (enhanced) green fluorescent protein; wPRE: woodchuck hepatitis virus post-transcriptional element.

To identify the structural attributes responsible for the anti-silencing function of the CBX3, we generated a total of six partially overlapping subfragments of the CBX3 element (Table 1, Fig. 1A). To generate cell populations representing a broad range of different integration sites, we employed lentiviral transduction. To this end, the subfragments were introduced into a third-generation self-inactivating (SIN)-lentiviral vector upstream of the viral SFFV promoter driving the expression of the eGFP reporter. Vectors employing the full length CBX3 (C-SG) and the 1.5 kb A2UCOE (A2-SG) served as positive controls, whereas the SFFV promoter alone (SG) was used as a negative control (Fig. 1B).

**Table 1:** Structural attributes of the A2UCOE, CBX3 and CBX3 subfragments.

**UCO elements**	**Length** (bp)	**CpG-sites** (#)	**G+C** (%)	**CpG density** (CpG/100 bp)
A2UCOE	1550	142	64.0	9.2
CBX3	679	63	67.9	9.3
CBX3(1-339)	339	18	57.6	5.3
CBX3(1-508)	508	35	62.2	6.9
CBX3(85-508)	424	31	63.9	7.3
CBX3(170-508)	339	26	66.7	7.7
CBX3(340-508)	168	17	71.4	10.1
CBX3(503-679)	177	28	83.1	15.8

bp, base pairs; #, number of

**Figure 2:**
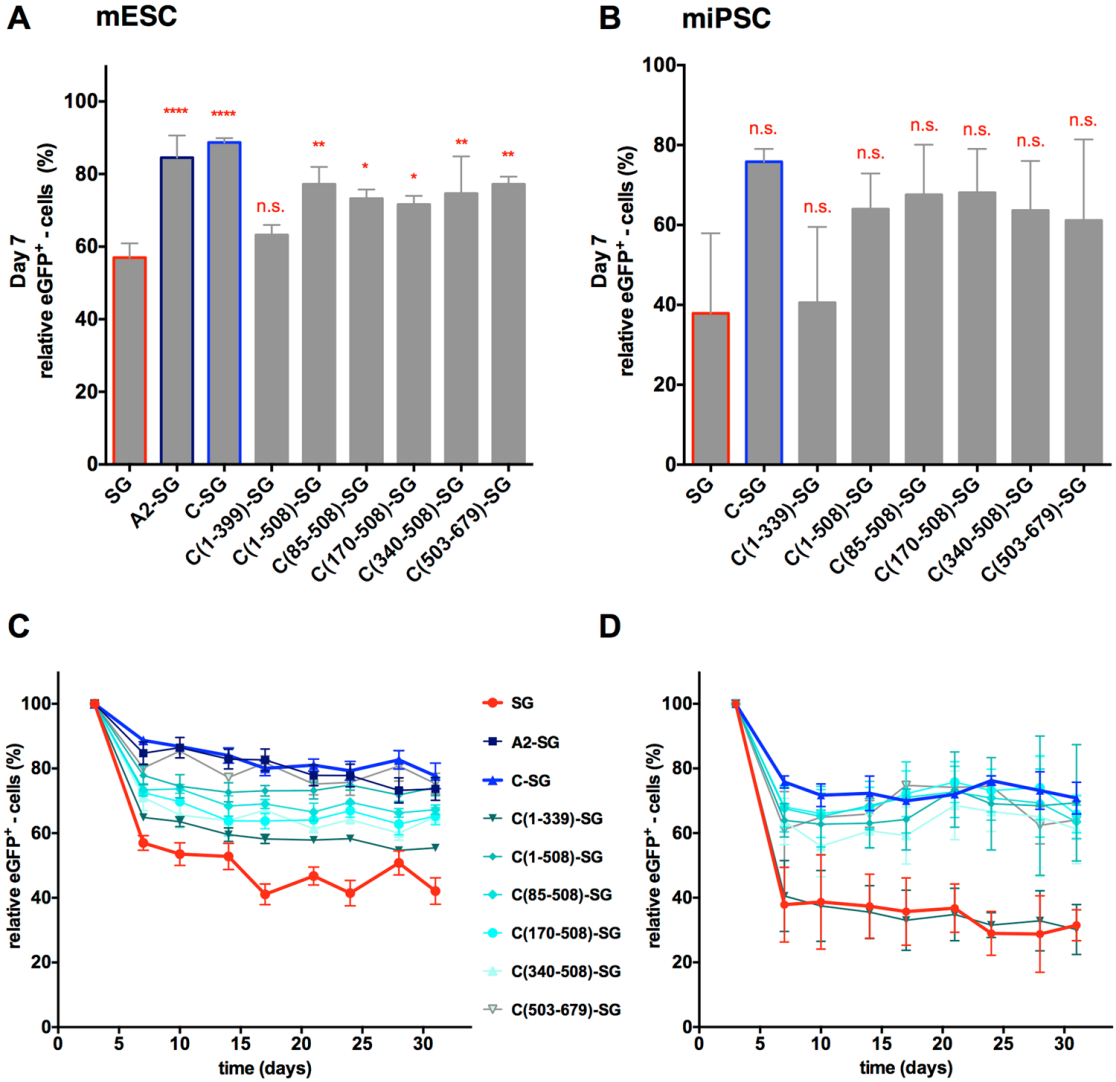
Activity of CBX3 subfragments in pluripotent stem cells. Murine ESCs and iPSCs were transduced with lentiviral vectors with or without the A2UCOE, the CBX3 or the CBX3 subfragments. The eGFP expression was monitored for 31 days and the relative expression (d3 = 100%) is shown for day 7 in (**A**) mESCs and (**B**) miPSCs. The time-course of relative eGFP expression for 31 days is depicted for (**C**) mESCs and (**D**) miPSCs. Activity of the subfragments was compared to the SG negative control. Data represent independent experiments n = 3; mean ± SD; *p < 0.05, **p < 0.01, ****p < 0.0001 as determined by one-way ANOVA.

A murine ESC as well as an induced pluripotent stem cell (miPSC) line were transduced with an MOI of 10 and 30, respectively. By this procedure, similar vector copy numbers (VCN) of approximately 2–3 for mESCs and 1–2 for miPSCs were achieved for all vectors (Supplementary Table S1). Expression of eGFP in pluripotent cells was monitored over a period of 31 days, i.e. 9 passages. Transduction efficiencies were assessed by flow cytometry on day 3 (Supplementary Fig. S1A) and served as a reference point to determine the relative number of eGFP-expressing cells over time. For all vectors and in both the mESC as well as the miPSC line, loss of transgene expression was primarily observed during the first 7 days (passage 2). This drop in transgene expression was more prominent for SG- than C-SG- or A2-SG-transduced control cells, indicating the transgene stabilizing function of the UCOEs. Cells transduced with the vectors C(1-508)-SG, C(85-508)-SG, C(170-508)-SG, C(340-508)-SG, or C(503-679)-SG maintained transgene expression at levels comparable to the positive controls ranging from 71% to 80% in mESCs and 61% to 68% in miPSCs (Fig. 2A,B). Only the C(1-339) fragment failed to display anti-silencing properties. Following this drop, the transgene expression stabilized and stable eGFP-expression was observed for at least 9 passages i.e. until day 31 (Figs 2C,D, S1B,C). Taken together, these results indicate particularly the CpG-rich central and 3′ region of the CBX3 region to be involved in UCOE-mediated transgene stabilization in pluripotent cells.

Biologic activity of the various fragments was also reflected by the median fluorescence intensity (MFI) of eGFP expression in transduced cell populations (Table 2), indicating the contribution of the CBX3-derived elements to the overall promoter activity. While the lowest MFI was observed for SG-transduced cells, at least in mESCs, C(1-339)-SG transduced cells displayed similar expression intensities as C(1-508)-SG, C(85-508)-SG, and C(170-508)-SG transduced cells. Lower expression intensities were observed particularly for C(340-508)-SG transduced cells in both mESCs and miPSCs.

**Table 2:** Expression intensity of CBX3 subfragments in pluripotent stem cells.

Median fluorescence intensity (MFI)				
Vector	mESC		miPSC	
	d3	d31	d3	d31
SG	54 ± 6	28 ± 4	26 ± 9	33 ± 1
A2-SG	201 ± 51***	142 ± 21****	-	-
C-SG	223 ± 25****	121 ± 17****	122 ± 61**	106 ± 38*
C(1-339)-SG	119 ± 27	104 ± 22***	37 ± 17	57 ± 6
C(1-508)-SG	148 ± 10*	128 ± 13****	75 ± 22	89 ± 12
C(85-508)-SG	142 ± 37*	135 ± 23****	89 ± 24	97 ± 39*
C(170-508)-SG	125 ± 37	113 ± 13***	75 ± 14	97 ± 16*
C(340-508)-SG	53 ± 17	43 ± 11	48 ± 11	48 ± 10
C(503-679)-SG	87 ± 4	56 ± 7	67 ± 6	81 ± 22

eGFP fluorescence mediated by the UCO elements was compared to the SG negative control. Data represent independent experiments n = 3; mean ± SD; *p < 0.05, **p < 0.01, ***p < 0.001, ****p < 0.0001 as determined by one-way ANOVA.

### Anti-silencing function of CBX3 subfragments in differentiated mESCs/iPSCs: correlation with CpG-sites

**Figure 3:**
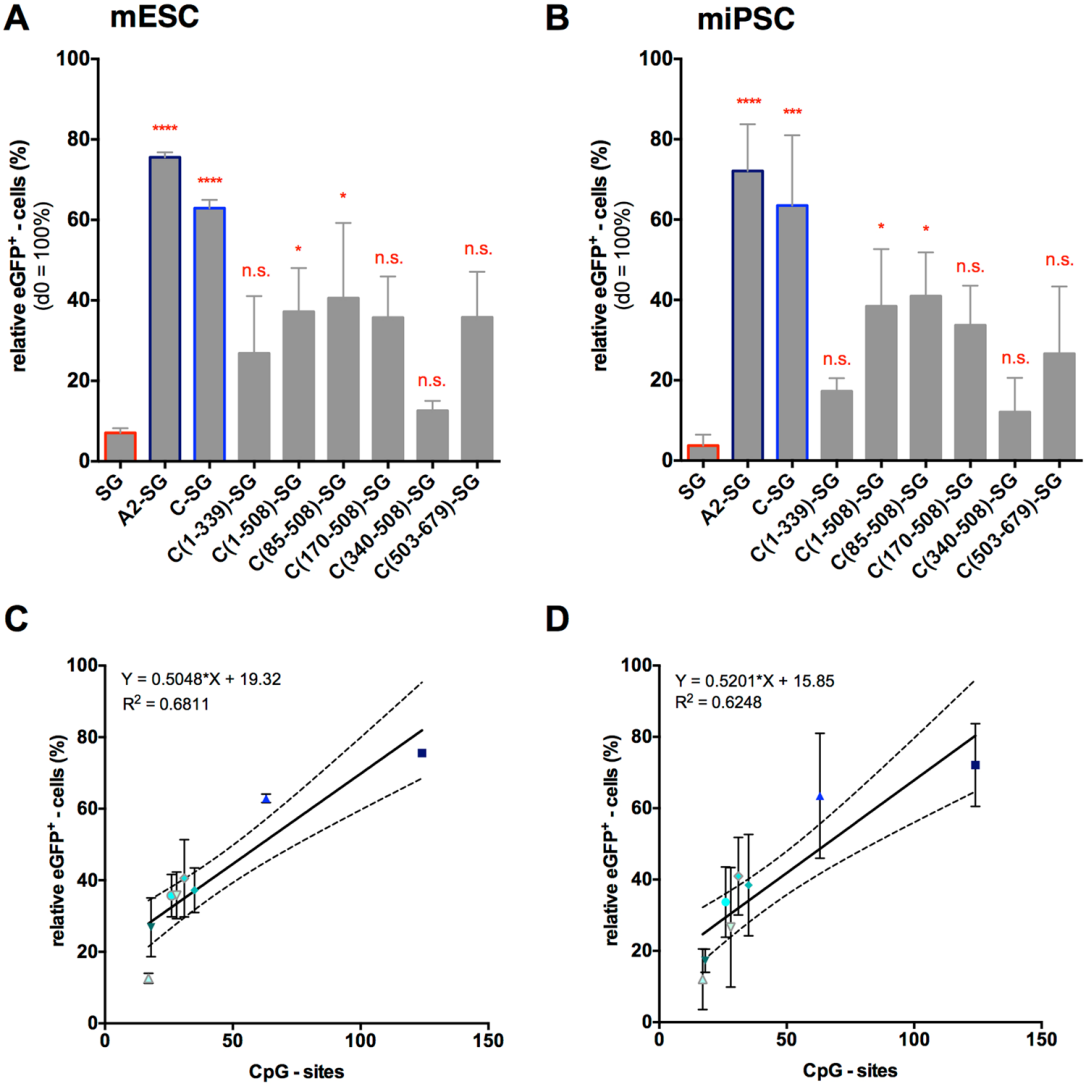
Activity of the CBX3 subfragments in differentiated cells. The transduced cells were subjected to an undirected EB-based differentiation and the eGFP expression was determined on day 0 as well as in SSEA-1 negative cells on day 8. The relative expression of eGFP was determined (d0 = 100%) for (**A**) mESCs and (**B**) miPSCs. (C,D) The correlation of relative eGFP expression in differentiated cells and the number of CpG-sites contained within each element is shown for (**C**) mESCs and (**D**) miPSCs (p < 0.0001). Activity of the subfragments was compared to the SG negative control. Data represent independent experiments n = 3; mean ± SD; *p < 0.05, **p < 0.01, ***p < 0.001, ****p < 0.0001 as determined by one-way ANOVA.

During differentiation stem cells undergo extensive epigenetic remodelling and euchromatic regions that served as favourable integration sites for lentiviral vectors may become heterochromatic, thereby posing a hindrance to lentiviral transgene expression^1, 2^. The *in vitro* differentiation of mESCs and miPSCs therefore represents a much more stringent system to analyse the potency of anti-silencing elements such as our CBX3 subfragments. To this end, the cells were subjected to undirected differentiation and the expression of eGFP in differentiated, SSEA-1 negative cells was assessed on day 8 (Fig. 3A,B). Whereas SG-transduced control mESCs (7%) as well as miPSCs (4%) showed a nearly complete loss of transgene expression upon differentiation, incorporation of CBX3 or A2UCOE into the vector markedly increased the population of transgene positive cells (CBX3: 63% (mESC), 81% (miPSC); A2UCOE: 64% (mESC), 72% (miPSC)). Also, all constructs with CBX3 subfragments stabilized transgene expression to some extent in differentiated mESC- and miPSC-derived cells when compared to SG controls, although these differences were significant only for CBX3(1-508) and CBX3(85-508). With eGFP-expressing cells ranging from 34% to 41%, however, neither of these subfragments showed full CBX3 activity. On the other hand, CBX3(1-339) and CBX3(340-508) clearly showed the lowest anti-silencing activity in both cell types. No major differences were observed between C(1-508)-SG, C(85-508)-SG, C(170-508)-SG and to some degree also C(503-679)-SG transduced cells. These data indicate that in addition to a central region common to the three subfragments with higher activity, also the 3′-end region of the CBX3 (bp 503 to bp 679) is involved in anti-silencing.

Interestingly, these four CBX3 subfragments all contain a high number of CpG-sites and the amount of CpG-sites per fragment was found to positively correlate with its anti-silencing capacity (measured as the percentage of eGFP-positive cells following differentiation) based on data for mESCs (R^2^ = 0.6811, p < 0.0001) as well as miPSCs (R^2^ = 0.6248, p < 0.0001) (Fig. 3C,D). While a similar correlation was observed for UCOE length (R^2^ = 0.6299, p < 0.0001 in mESCs; R^2^ = 0.5848, p < 0.0001 in miPSCs), no significant correlation was detected for CpG-site density (CpGs/100 bp) or GC-content (data not shown).

### Functionality of a scrambled CBX3-UCOE in pluripotent and differentiated mESCs/miPSCs

**Figure 4:**
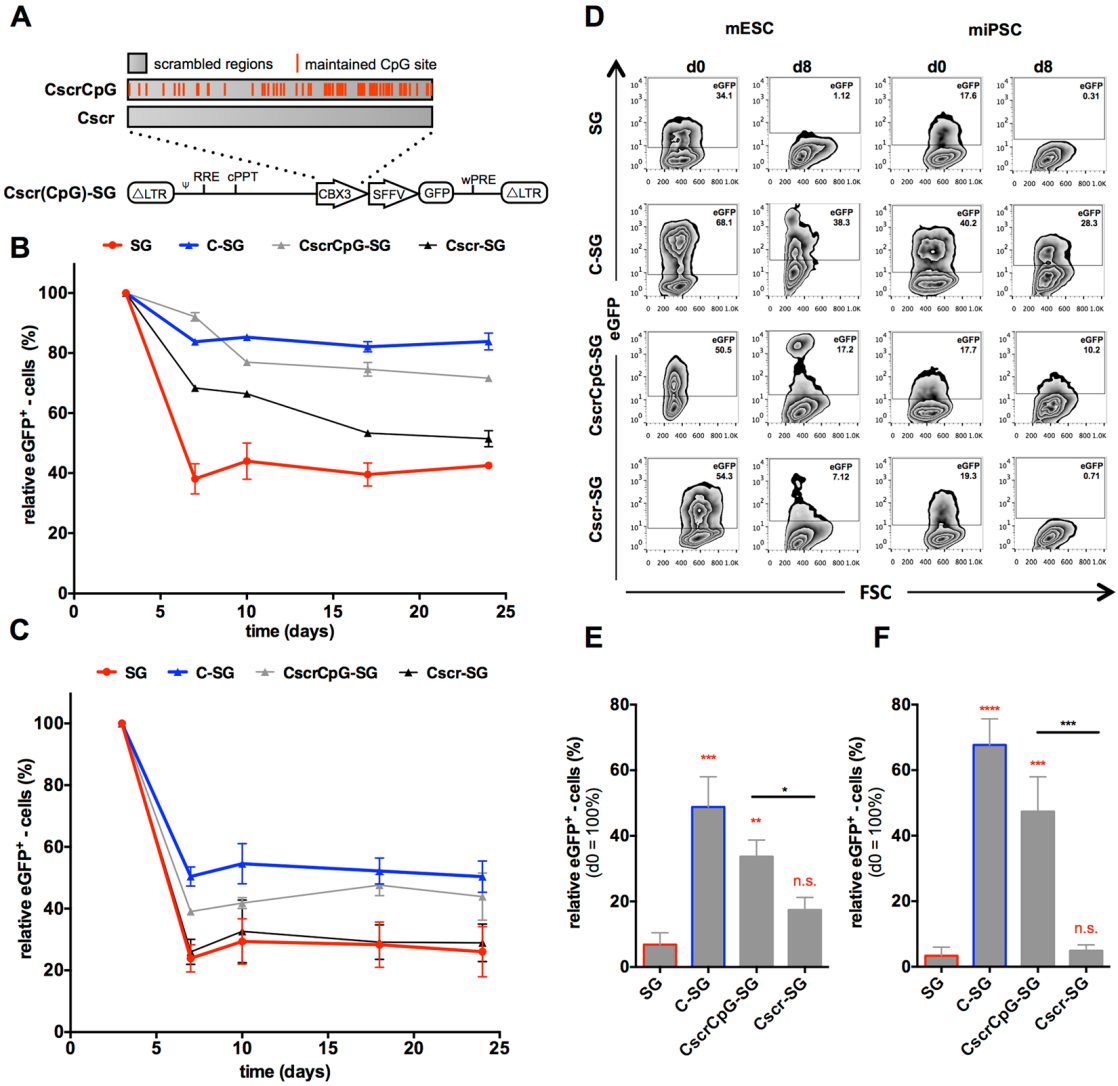
Anti-silencing activity of synthetic scrambled CBX3 elements. The DNA sequence of the CBX3 was shuffled randomly to create two synthetic elements that harbor the same nucleotide composition in a random order with one sequence maintaining the CpG-sites at their original position (CscrCpG) and the other completely destroying these sites (Cscr). (**A**) The elements were introduced into the SFFV.eGFP (SG) third generation SIN-lentiviral vector upstream of the promoter. (**B**,**C**) Following transduction, the eGFP expression was monitored by flow cytometry for 24 days in SSEA-1 positive (**B**) mESCs and (**C**) miPSCs. (**D–F**) The transduced cells were subjected to an EB-based undirected differentiation with transgene expression measured on day 0 and in SSEA-1 negative cells on day 8. (**D**) Representative flow cytometric analysis of mESCs and miPSCs prior to (d0) and after differentiation (d8). Relative eGFP expression was determined in differentiated (SSEA-1 negative) (**E**) mESCs and (**F**) miPSCs. Activity of the synthetic elements was compared to the SG negative control. Data represent independent experiments n = 3; mean ± SD; *p < 0.05, **p < 0.01, ***p < 0.001, ****p < 0.0001 as determined by one-way ANOVA.

To further assess the relevance of the CpG-sites within the CBX3 element, two scrambled versions of the CBX3 were designed. The first element was generated by random shuffling of the DNA sequence while retaining the CpG-sites (roughly 20% of total sequence) at their original position without changing the overall nucleotide composition (CscrCpG). The second element was designed by randomly shuffling the complete DNA sequence thereby destroying the CpG-sites (Cscr; alignment of sequences given in Supplementary Fig. S2). Again, the elements were inserted into the SG lentiviral vector thus giving rise to the constructs CscrCpG-SG and Cscr-SG (Fig. 4A).

Again, mESCs and miPSCs were transduced while aiming for comparable transduction efficiencies and VCNs (Supplementary Table S3). The eGFP expression in pluripotent cells was monitored by flow cytometry for a period of 24 days (Fig. 4B,C). In agreement with our previous data, loss of eGFP expression was primarily observed up to day 7 (passage 2) with relatively constant numbers of eGFP-positive cells thereafter. After 24 days, only the CscrCpG-SG transduced mESCs and miPSCs displayed significantly more eGFP-positive cells (72% and 44%) than SG-transduced negative controls (43% and 26%). In contrast, no significant differences to the negative control were identified in both Cscr-SG transduced mESCs (51%) and miPSCs (29%) after 24 days (Supplementary Fig. S3A,B). Of note, only C-SG transduced cells demonstrated an increase in MFI above SG levels (Supplementary Table S3), whereas neither the incorporation of CscrCpG nor Cscr could achieve this.

To assess the anti-silencing activity of the scrambled CBX3 elements during differentiation, we determined the relative eGFP expression in SSEA-1 negative cells following eight days of undirected differentiation using day 0 as reference (Fig. 4D–F). Confirming our previous data, SG transduced mESCs and miPSCs lost most of their expression upon differentiation (down to 8% and 3%, respectively), whereas C-SG transduced mESCs and miPSCs demonstrated significantly higher levels of eGFP expressing cells (48% and 68%, respectively). Interestingly, also CscrCpG-SG cells showed significantly elevated levels of transgene expression upon differentiation of both mESCs (33%) and miPSCs (47%). Cscr-SG transduced cells, on the other hand, failed to show significant anti-silencing activity in this assay with transgene expression in mESC- or miPSC-derived cells of only 17% or 4%, respectively. These results further highlighted the importance of the CpG-sites for the anti-silencing function of the CBX3.

### Promoter activity of CBX3-UCOE fragments in pluripotent and differentiated mESCs/miPSCs

**Figure 5:**
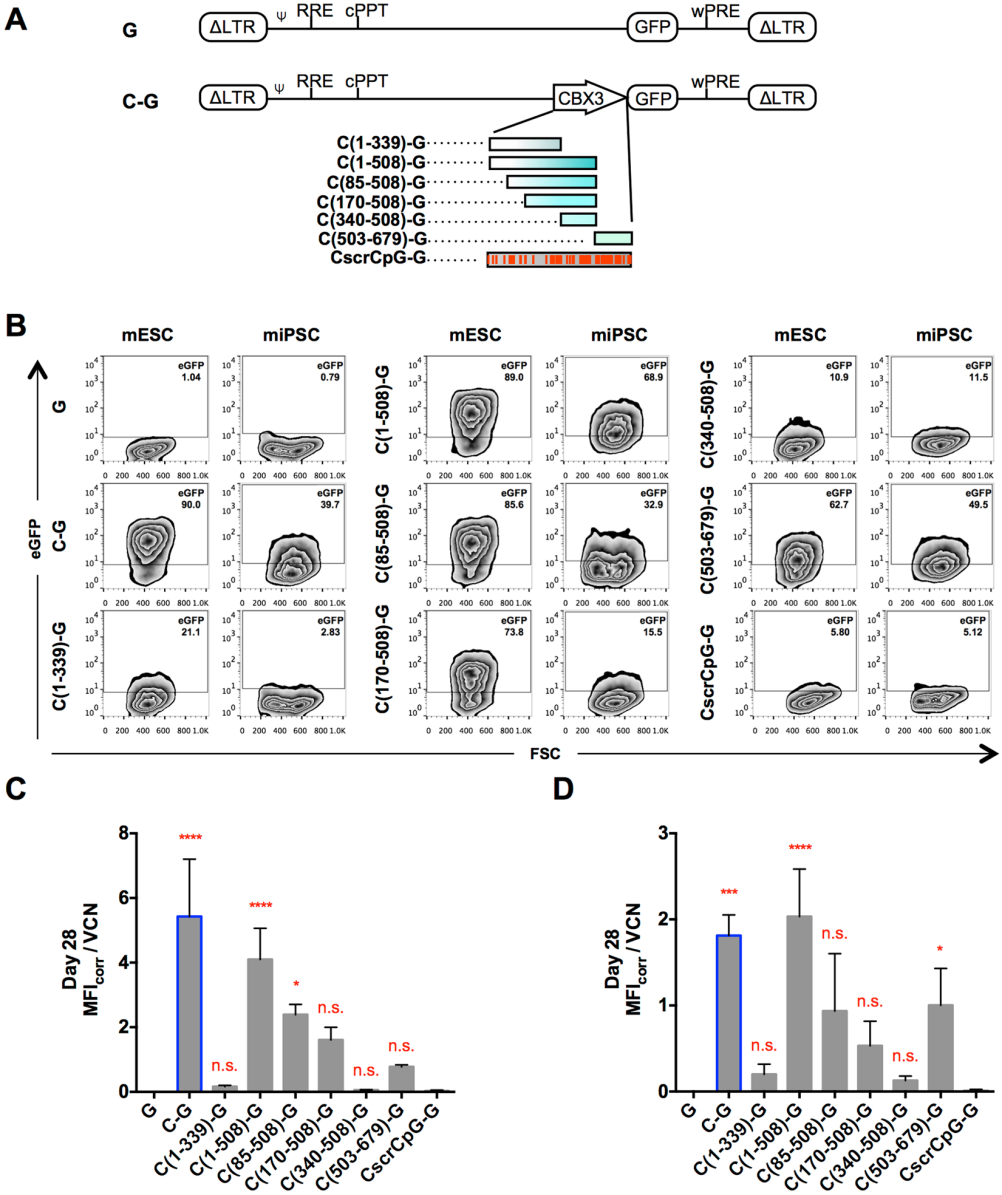
Promoter activity of CBX3 derived fragments. (**A**) Third generation SIN-lentiviral vectors harboring an eGFP cDNA preceded by CBX3-derived elements. (**B**) Representative flow cytometric analysis of eGFP expression in mESCs and miPSCs three days after transduction. (**C-D**) Median fluorescence intensity (MFI) of eGFP-expressing cells was measured by flow cytometry, corrected for background eGFP expression (MFI_corr_; G = 0) and determined per vector copy number (VCN) in pluripotent (SSEA-1 positive) (**C**) mESCs and (**D**) miPSCs 28 days after transduction. Activity of the subfragments was compared to the eGFP negative control. Data represent independent experiments n = 3; mean ± SD; *p < 0.05, ***p < 0.001, ****p < 0.0001 as determined by one-way ANOVA.

The data given above on MFI for eGFP expression suggest that both the A2UCOE and the CBX3 still harbour intrinsic transcriptional activity. The dual promoter activity of the A2UCOE, however, seems to be dispensable for its anti-silencing activity, since the CBX3 with its single promoter is still highly efficient in this respect. On the other hand, another 0.7 kb UCOE derived from the first intron of CBX3 but lacking intrinsic transcriptional activity was shown to mediate only partial protection from silencing of the SFFV promoter *in vitro* ^18^. Taken together with the observation that the A2UCOE showed an orientation-dependent anti-silencing effect with improved function only when the CBX3 promoter was facing the juxtaposed heterologous promoter^11^, this poses the question whether intrinsic promoter activity in the direction of the transgene has a role in the anti-silencing ability of the UCOEs. Since the CBX3 is a CpG-island promoter and thus does not contain known defined motifs, such as a TATA box, that could be mutated to erase the promoter function, we investigated the promoter activity of the CBX3 subfragments instead and compared this to their anti-silencing ability. For these studies, the SFFV promoter was excised from the SG lentiviral vectors, giving rise to the vector constructs depicted in Fig. 5A.

Three days following the transduction of mESCs and miPSCs, the eGFP expression was assessed by flow cytometry **(**Fig. 5B). Since for some constructs it was difficult to determine the titre of the lentiviral supernatants due to low or no eGFP expression, some variability of VCN could not be avoided (Supplementary Table S4). As expected, right after transduction (day 3) the eGFP control construct (G) showed only a minimal background MFI for mESCs as well as miPSCs. Minimal to no promoter activity was also observed for the subfragments C(1-339), C(340-508) as well as the synthetic element CscrCpG. In contrast, C(1-508), C(85-508), C(170-508), and C(503-679) clearly demonstrated endogenous promoter activity, which for some constructs was similar to the activity of CBX3 that was used as the positive control.

To facilitate the read-out, for further analysis the background MFI of G-transduced cells was deducted from all MFIs measured in the same experimental setting yielding MFI_corr_. Moreover, to account for the differences in VCN, the MFI_corr_ per VCN (MFI_corr_/VCN) was calculated and used for further analysis. When MFI_corr_/VCN was monitored over a 28-day period, relatively stable eGFP expression levels were observed for the individual constructs (Supplementary Fig. S4A,B) and even on day 28 significant promoter activity was detected for the C(1-508) subfragment in both mESCs and miPSCs as well as for the C(85-508) in mESCs and the C(503-679) in miPSCs. Although not significant, C(170-508) also showed promoter activity in both mESCs and miPSCs (Fig. 5C,D). Of note, despite its anti-silencing activity in the context of SG lentiviral vectors, the CscrCpG showed almost no eGFP expression in these experiments, indicating that the mediated anti-silencing effect was independent of transcriptional activity. In contrast, the other fragments that previously demonstrated good anti-silencing ability in the context of SG-based lentiviral vectors, i.e. C(1-508), C(85-508), C(170-508), and C(503-679), exhibited notable promoter activity in mESCs as well as miPSCs. According to these data, for the CBX3 subfragments higher endogenous promoter activity tended to correlate positively with their ability to protect SG-based vectors from silencing in mESCs (p < 0.0001) or miPSCs (p = 0.0007) (Supplementary Fig. S4C,D). Little endogenous promoter activity was, however, observed for all constructs except for the complete CBX3 element following eight days of directed differentiation (Supplementary Fig. S4E,F).

### CBX3 function at defined chromosomal loci

**Figure 6:**
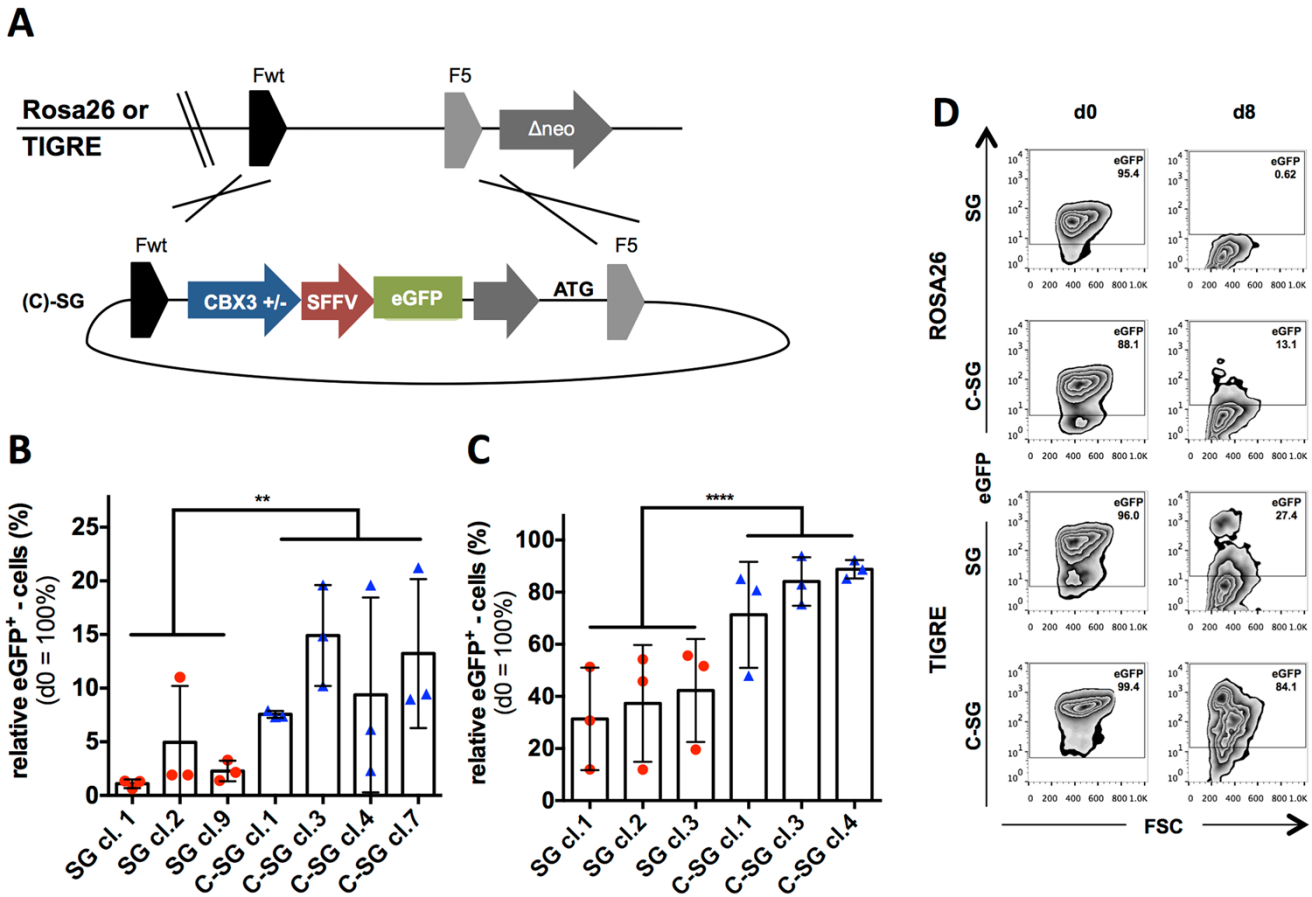
Anti-silencing function of the CBX3 at two defined chromosomal loci. (**A**) Murine ESCs were targeted at either the ROSA26 or the TIGRE chromosomal locus by using FLP-mediated cassette exchange to introduce either an SFFV.eGFP (SG) or CBX3.SFFV.eGFP (CSG) cassette. Subclones of (**B**) ROSA26- (SG: 3 clones; CSG: 4 clones) and (**C**) TIGRE- (SG: 3 clones; CSG: 3 clones) targeted cells were subjected to EB-based undirected differentiation and the relative percentage of eGFP-expressing cells was determined by flow cytometry on day 8 in SSEA-1 negative cells (d0 = 100%). (**D**) Representative flow cytometric analysis of targeted cells on day 0 and day 8. Bar charts represent mean ± SD; n = 3 per clone. Student’s t-test was performed on pooled SG-clones (ROSA26, n = 9; TIGRE, n = 9) and C-SG clones (ROSA26, n = 12; TIGRE, n = 9); **p < 0.01, ****p < 0.0001.

Lentiviral transduction of stem and progenitor cells is accompanied with genome-wide integration of vectors. This was described to result in heterogeneous transgene expression in individual cell clones of cell populations^9–12, 19^. It was shown that transduction with UCOE-containing vectors resulted in significantly less heterogeneity than populations of cells with UCOE-free vectors^9, 10, 15^. However, it was not clear whether the UCOE-mediated protection from epigenetic modulations was exhibited equally at all integration sites or only as a consequence of integration into chromosomal sites with favourable epigenetic features. To address this notion, we used Flp recombinase mediated cassette exchange (RMCE) to investigate the impact of a UCOE at two defined chromosomal sites. In particular, we integrated a cassette comprising the silencing prone viral SFFV promoter driving eGFP with or without the CBX3 UCOE at two defined chromosomal loci in mouse embryonic stem cells (mESCs); the ROSA26 and the TIGRE locus (Fig. 6A). The incorporation of the eGFP reporter allowed single cell analyses to visualise the heterogeneous effect of transgene silencing. In the ROSA26 locus of pluripotent mESCs the percentage of transgene expressing cells gradually decreased to 60-80% for all isogenic SFFV.eGFP (SG) as well as all CBX3.SFFV.eGFP (C-SG) clones (Supplementary Fig. S5A), whilst the expression of eGFP remained fairly stable over 6–10 consecutive passages at the TIGRE locus for both SG and C-SG clones (Supplementary Fig. S5B). The comparable loss of eGFP expression at ROSA26-targeted isogenic subclones indicates similar epigenetic silencing kinetics upon RMCE, which suggests the presence of a highly reproducible modification.

To assess the CBX3 function during differentiation, the transgene expression was determined for both ROSA26 and TIGRE targeted cells (Fig. 6B,C) on day 0 and day 8 of an undirected EB-based differentiation. Here, day 0 served as reference point to determine the relative number of eGFP-expressing cells after eight days of differentiation. The SG clones of ROSA26 targeted cells showed nearly a complete loss of transgene expression with only 1–5% eGFP-positive cells remaining. Incorporation of the CBX3 increased eGFP-positive cells to 7–15% (Fig. 6B). At the TIGRE locus, undirected differentiation resulted in 31–42% eGFP-expressing cells in SG clones, which was increased to 71–89% eGFP-positive cells in C-SG clones (Fig. 6C). Representative flow cytometric analyses are shown in Fig. 6d. Taken together, while different levels of epigenetic repression were observed in these two defined chromosomal sites, the CBX3 was able to significantly stabilise transgene expression at both loci. This proves that UCOEs can overcome epigenetic restrictions in defined chromosomal sites, thereby justifying the use of bulk populations to investigate UCOE functions.

## Discussion

UCOEs such as the A2UCOE have been demonstrated to efficiently negate epigenetic repression of transgene expression from retroviral vectors in a variety of cell lines and primary cells including hematopoietic stem cells as well as murine and human pluripotent ESCs and iPSCs and their differentiated progeny^9–11, 20^. This protective activity has been linked with reduced promoter CpG methylation as well as decreased levels of repressive and increased levels of active histone marks in the chromosomal neighbourhood of UCOEs. While traditionally bidirectional promoter activity has been deemed a necessary trait for the anti-silencing function of UCOEs, we recently described the 0.7 kb CBX3 element that lacks the HNRPA2B1 promoter moiety of the A2UCOE but still potently stabilizes lentiviral transgene expression in multipotent and pluripotent stem cells^15^. Thus, the CBX3 represents a unique single-promoter UCOE that still shows most if not all of the anti-silencing activity of the A2UCOE and due to its small size it does not adversely affect viral titres^15^.

Although the CBX3 has been shown to protect transgene expression from viral, housekeeping and tissue-specific promoter elements, a link of this activity to distinct structural features of the CBX3 is still missing. To address this point, we investigated the anti-silencing ability of various CBX3 subfragments. While all subfragments except for the 5′ CBX3(1-339) fragment showed substantial anti-silencing activity in the pluripotent state, in differentiated cells two subfragments, CBX3(1-339) and CBX3(340-508), failed to show anti-silencing activity. The remaining subfragments CBX3(1-508), CBX3(85-508), CBX3(170-508) and CBX3(503-679) all showed activity in pluripotent as well as differentiated cells. These data indicate different mechanisms to be responsible for transgene silencing in the pluripotent state or during cellular differentiation. While transgene silencing in the pluripotent state is well documented^1, 9, 10, 21^, also the extensive chromatin remodelling associated with the exit from pluripotency has been advocated as a factor that impairs retroviral transgene expression. In this context, loss of transgene expression in differentiated cells has been described by a number of groups^6, 22^, and this notion is further supported by previous data from our group describing profound transgene silencing as well as anti-silencing efficacy of the A2UCOE particularly during the early differentiation state of ESCs and iPSCs when cells leave pluripotency^9, 10, 15^.

Of note, our data imply that the anti-silencing function of the CBX3 element cannot be mapped to a single region and thus emphasize the CBX3 element as a minimal UCOE version with almost complete functionality of the original 1.5 kb A2UCOE. While all subfragments displayed at least some level of activity, none of the fragments could completely substitute for the CBX3 in our experimental models. Nevertheless, the considerable activity of the CBX3(503-679) and the three subfragments containing the 170-508 bp sequence indicate the relevance of the central as well as the 3′ portion of the CBX3 in this context. In contrast, the rather low activity of the CBX3(1-339) subfragment and the similar properties of the CBX3(1-508), CBX3(85-508), and CBX3(170-508) subfragments suggest only moderate contribution, if any at all, from the 5′ end of the CBX3. We have not been able, however, to generate CBX3 subfragments lacking only the 5′ 170 or 339 bp to more formally test this hypothesis. Importantly, we demonstrated a significant correlation of fragment length and, even more important, overall CpG content with anti-silencing activity, further supporting the notion that the highly CpG-dense central and 3′ regions may be crucial determinants of CBX3 function.

The CpG-islands within the A2UCOE have long been considered a central component of the anti-silencing function. Indeed, we now show that a scrambled CBX3 element (CBX3scrCpG) that bears no resemblance to the CBX3 (or any other UCOE) besides maintained CpG-sites, has the ability to protect lentiviral transgene expression in pluripotent cells and thereof differentiated progeny. A completely scrambled CBX3 variant (CBX3scr) lacking the CpG-sites displayed minimal to no functionality. Although not completely equivalent in functionality to the CBX3 element, this ability of the CBX3scrCpG is quite remarkable, and strongly indicates the CpG-sites of the CBX3 as important structural determinants of its anti-silencing function. CpG-islands are detected in the proximity of 60-70% of gene promoters^23, 24^, and their non-methylated CpG-sites have been identified as specific targets of various histone modifying proteins as well as DNA demethylating enzymes (reviewed in ref. 25). All of these proteins including amongst others Cfp1 (CxxC finger protein 1), Mll (mixed lineage leukemia protein), or Kdm2a (H3K36 demethylase) share a common feature, a zinc finger CxxC binding domain that specifically recognizes and binds non-methylated CpG-sites^26^. Specifically Cfp1 has been shown to associate with non-methylated CpGs both *in vitro* and *in vivo* ^27^ and to interact with the H3K4 methyltransferase Setd1^28, 29^, thereby leading to the deposition of the active chromatin mark H3K4me3. Interestingly, artificial, promoter-less, non-methylated CpG-stretches were able to recruit Cfp1 and create new H3K4me3 marks when integrated into the genome of mouse ESCs^27^. Thus, future studies that investigate the physical interaction of CBX3 with such CxxC-domain containing proteins may help to shed light on the molecular mechanism by which the CpG-islands of the CBX3 mediate its anti-silencing function.

Moreover, regions highly enriched in non-methylated CpG-dinucleotides (CpG islands) are associated with an open chromatin structure that particularly in pluripotent cells is marked by a bivalent chromatin containing both active as well as repressive chromatin marks^30^. Synthetic CpG-islands have been shown to induce this bivalent state in ESCs if the synthetic sequence in addition to the CpG-dinucleotides is also enriched for G and C nucleotides^31^. This may imply that in addition to the CpG-sites also the overall GC-density of the CBX3 and thereof derived subfragments supports the overall function of these elements.

Considering the impact of transcriptional activity on the chromatin status and the recruitment of binding factors, such as Cfp1, in the vicinity of CGIs, it becomes evident that the promoter activity of CBX3 may actually constitute a relevant factor for its anti-silencing function. In support of this notion we observed that CBX3 subfragments with higher endogenous promoter activity also tended to perform better with respect to their anti-silencing function. Along the same line, in the hands of other investigators a different CBX3 element devoid of transcriptional activity (0.7 kb intronic UCOE) displayed poor anti-silencing activity^18^. On the other hand, the anti-silencing ability of the CBX3scrCpG element that lacked transcriptional activity indicates that CpG-site mediated anti-silencing can be independent of intrinsic promoter activity. However, since the synthetic element was less effective than the CBX3, it can be speculated that a combination of CpG motives and promoter activity might be important for full anti-silencing activity of the CBX3.

Additionally, the CBX3(340-508) and the CBX3(1-339) subfragments, while sharing a low number of CpG-sites and a low promoter activity, differ markedly in their anti-silencing potential specifically in the pluripotent state. This clearly indicates the contribution of additional and as of yet undefined factors, such as for example the presence of specific transcription factor binding sites, to the anti-silencing potential of the CBX3.

Up to now, most studies on UCOE functionality have been performed in polyclonal cell populations, which are characterized by a random panel of integration sites. So far, studies that allow the evaluation of UCOEs in a defined chromosomal context are lacking. Thus, it is not known if UCOEs can stabilize expression in all chromosomal sites prone to silencing and if they do that to a similar extent. To directly compare expression properties in the presence of absence of an UCOE, we now analysed the activity of the CBX3 at two defined chromosomal loci in mESCs; the ROSA26 and the TIGRE locus. The two loci were chosen because of their expression properties: The endogenous Rosa26 promoter is active in cells of all differentiation states^16^; upon integration of heterologous promoters, variable expression patterns were observed suggesting that some of the promoters undergo substantial silencing^32, 33^. The TIGRE locus was identified upon screening for sites supporting tightly regulated transgene expression^17^ in a broad range of tissues suggesting an euchromatic state.

To facilitate efficient targeting of transgene cassettes into these sites we employed Flp recombinase mediated cassette exchange (RMCE). This method has been utilized for direct comparison of vector designs at defined chromosomal sites^34–36^, thereby excluding the varying influence of random chromosomal sites.

Silencing of the SFFV cassette in the Rosa26 locus is in line with previous studies indicating the loss of transgene expression from various promoters in this chromosomal locus^32, 33^. Interestingly, silencing of the SFFV promoter was less pronounced in the TIGRE locus. Of note, incorporation of the CBX3 into the transgene expression cassette increased the percentage of eGFP-retaining cells after differentiation in both loci, supporting the broad applicability of the CBX3.

Transgene silencing at the ROSA26 locus demonstrated a gradual reduction of eGFP expression even in pluripotent cells despite the presence of the CBX3, and upon differentiation only a moderate, though significant, activity of the CBX3 was noted. This clearly indicates that the UCOE cannot completely shield the incoming cassettes from negative influences of the ROSA26 locus making this locus an unsuited site for integration of silencing prone promoters. Recently, another group evaluated various promoters upon RMCE based targeting into the AAVS1 safe harbour locus in hESCs. Interestingly, depending on the promoter, they encountered variable levels of transgene inhibition, both in pluripotent cells and during hepatic differentiation^37^. These findings confirm our previous notion that not the chromosomal site as such but rather the combination of integration site and incoming promoter defines the expression properties^38^. Thus, the concept of universally ‘safe’ loci with predictable expression may need to be revised and assessed more carefully. In contrast to the ROSA26 locus, the TIGRE locus was more permissive to transgene expression particularly in pluripotent cells. Also this locus notably repressed the SFFV driven transgene expression during differentiation but the incorporation of a CBX3 resulted in significant improvement, rescuing expression in about 80% of cells. These data thus demonstrate that the CBX3 is able to alleviate transgene silencing at both the ROSA26 and the TIGRE locus, albeit at different levels.

Taken together, our data indicate that the CpG-sites as well as the transcriptional activity significantly contribute to the anti-silencing function of the CBX3. Moreover, given the differential activity observed for individual subfragments in the pluripotent state and during differentiation, as well as the anti-silencing properties observed for the CscrCpG element despite its lack of transcriptional activity, our data suggest that different mechanisms are involved in the process of CBX3-mediated transgene stabilisation.

## Materials and Methods

### Generation of CBX3 subfragments (PCR and restriction digest)

To generate the CBX3 subfragments, specific primers were designed and modified to include either an XhoI restriction site (forward) or NheI restriction site (reverse) (Supplementary Table S5). All subfragments, except CBX3(503-679), were generated by using a temperature gradient PCR protocol that included the combination of the respective forward and reverse primers (Supplementary Table S5), Phusion High-Fidelity DNA polymerase (ThermoFisher Scientific), and up to 7% DMSO. Subsequently, the CBX3 subfragments were digested with XhoI and NheI restriction enzymes and cloned into a third-generation SIN-lentiviral vector upstream of the spleen focus forming virus (SFFV) promoter (pRRL.PPT.SFFV.eGFP.pre* ^39^; referred to as SFFV.eGFP) to generate the vectors pRRL.PPT.CBX3(bp-bp).SFFV.eGFP.pre* referred to as CBX3(bp-bp).SFFV.eGFP. Insertion of the CBX3 element into the SFFV.eGFP by using the restriction enzymes XhoI and NheI gave rise to the vector pRRL.PPT.CBX3.SFFV.eGFP.pre* (CBX3.SFFV.eGFP). The CBX3(503-679) subfragment was generated by digesting the CBX3.SFFV.eGFP with XhoI and ApaI, filling the ends by using Klenow fragment (ThermoFisher Scientific) and subsequently self-ligating the vector to generate the construct CBX3(503-679).SFFV.eGFP.

### Vector production

Lentiviral supernatants were produced by transient co-transfection of 293 T cells using calcium phosphate precipitation according to the previously described protocol^40^. In brief, 7 × 10^6^ 293 T cells were cultured on 10 cm dishes in high-glucose Dulbecco’s Modified Eagles Medium (DMEM) supplemented with heat-inactivated foetal calf serum (FCS,10%), penicillin/streptomycin (1 mM) and L-glutamine (1 mM) (all Invitrogen). The cells were transiently co-transfected with lentiviral vector (5 µg), pcDNA3.GP.4xCTE (expressing the HIV-1 gag/pol, 8 µg), pRSV-Rev (5 µg), and pMD.G (carrying the vesicular stomatitis virus glycoprotein, 2 µg) in the presence of HEPES buffer (ThermoFisher Scientific) and chloroquine (Sigma Aldrich). 48 hours and 72 hours after transfection viral supernatants were harvested and concentrated by ultracentrifugation at 4 °C. Subsequently, viral titres were determined by transduction of the murine SC-1 fibroblast cell-line (low-glucose DMEM, 10% FCS, 1 mM penicillin/streptomycin, 1 mM L-glutamine, and 4 µg/mL protamine sulfate) in serial dilution and flow cytometric analysis of eGFP-expressing cells 3 days thereafter.

### Murine pluripotent stem cell cultivation

The mESC line CCE^41^, the miPSC line CD45.1^42^ as well as the RMCE based mESC clones were cultured on mitomycin-C treated murine embryonic fibroblast (MEF) feeder cells in ESC medium (Knock-out DMEM, 15% ES-tested FCS, 1 mM L-glutamine, 1 mM non-essential amino acids, 1 mM penicillin/streptomycin, 0.05 mM β-mercaptoethanol and 10^3^ U/mL leukaemia inhibitory factor (LIF) (kindly provided by the Institute of Technical Chemistry, Hannover Medical School)). The medium for RMCE based mESCs was additionally supplemented with CHIR (99021, 1.5 µM) and PD (0325901, 0.5 µM) to maintain their pluripotency. The cells were passaged every 2–3 days using Trypsin (Invitrogen).

### Transduction

For transduction, 1 × 10^5^ mESC or miPSC single cells were seeded per well on gelatine-coated 12-well plates containing standard ESC-medium supplemented with protamine sulfate (Sigma Aldrich, 4 μg/mL) and lentiviral supernatant was added (MOI 10 or 30 (based on the viral titres determined on SC-1 cells) for mESC or miPSC, respectively). 3–4 days thereafter the transduced cells were transferred onto MEF feeder cells with fresh medium and cultured as described above.

### Targeting of ROSA26 and TIGRE locus in mESC and clonal expansion

The targeting of the ROSA26 chromosomal locus was performed by Flp recombinase mediated cassette exchange (RMCE) in G4B12 cells^43^, which harbour an RMCE compatible FRT-wt/FRT-F5 flanked expression cassette as well as a silent neomycin phosphotransferase expression unit at the ROSA26 locus^43^. RMCE compatible TIGRE ES cells carry a single copy of chromosome 19 derived BAC RP24-481E2 which was genetically engineered by integrating an RMCE-compatible FRT-wt/FRT-F5 flanked CMV-driven GFP cassette as well as a silent neomycin phosphotransferase expression unit in the tightly regulated (TIGRE) T1 locus^17^, which was designated as the TIGRE locus in this study. RMCE was induced in either G4B12 or TIGRE ESCs by co-transfection with FRT-wt/FRT-F5 flanked SFFV.eGFP or CBX3.SFFV.eGFP containing plasmids together with an FLP recombinase expression plasmid. G418-mediated selection was used to isolate correctly targeted isogenic subclones which were expanded individually^44^.

### Undirected differentiation of mESC/miPSC

Undirected differentiation was carried out as previously described for hematopoietic differentiation^45^ but without the addition of cytokines on day 5. In brief, mESCs or miPSCs were seeded on gelatine-coated 6-well or 12-well plates and cultured for 2–3 days without feeders. The cells were then moved into suspension culture to allow embryoid body (EB) formation. On day 8 the cells were harvested, dissociated using Collagenase IV (STEMCELL technologies), stained for SSEA-1 expression and analysed by flow cytometry.

### Flow cytometry

Pluripotent as well as differentiated mESCs and miPSCs were stained for stage specific-antigen embryonic antigen-1 (SSEA-1) expression using the Anti-Human/Mouse SSEA1-APC (eBioscience) antibody. Flow cytometric analysis was performed as described^40^. In brief, cells were harvested and dissociated as described above and resuspended in phosphate buffered saline (PBS) (Invitrogen). The cells were then stained, washed with FACS buffer and analysed with a FACScalibur machine (Beckton & Dickinson). The raw data were then analysed with the software FlowJo (TreeStar).

### Quantitation of vector copy number (quantitative real-time PCR)

Genomic DNA was isolated from transduced mESCs and miPSCs using the GenElute Mammalian Genomic DNA Miniprep Kit (Sigma-Aldrich). The vector copy number (VCN) was determined by quantitative PCR as was previously described^46, 47^. In brief, samples were analysed for the presence of the woodchuck post-transcriptional element (wPRE) and the polypyrimidine tract binding protein 2 gene (ptpb2; as reference). A linear plasmid containing both the wPRE and the ptpb2 sequence was used to generate a standard curve.

### Design of synthetic scrambled CBX3-UCOE

The current study used the splice-site deleted version of the CBX3 element (originally termed CBX3* ^15^). The CBX3 sequence was randomly shuffled by using the DNA shuffling tool from the bioinformatics homepage (http://www.bioinformatics.org/sms2/shuffle_dna.html). To obtain the scrambled CBX3 with preserved CpG-sites (CscrCpG), the CpG-sites were extracted from their original position in the CBX3 sequence. The remaining sequence was then shuffled and the CpG-sites were subsequently reintroduced at their original position. The resulting sequence was then modified by shuffling specific areas to remove any newly formed CpG-sites, any identified start codons (ATG; lack of open reading frames was confirmed by using the open reading frame (ORF) finder from bioinformatics; http://www.bioinformatics.org/sms2/orf_find.html), as well as any recognition sites for the restriction enzymes XhoI and NheI (needed for subsequent cloning). The final sequence was analysed using the National Center for Biotechnology Information (NCBI) Nucleotide BLAST tool and no similarities to other sequences were identified. To obtain the fully scrambled CBX3 element, the same procedure was used without previously extracting the CpG-sites. Both of these scrambled CBX3 elements were then synthesized by Eurofins Genomics and subsequently cloned upstream of the SFFV promoter in an SFFV.eGFP third-generation SIN-lentiviral vector.

### Statistics

The software GraphPad Prism version 6 (Graphpad Software Inc) was applied to perform Student’s t-test (α = 0.05) for a comparsion of two groups or one-way ANOVA with Tukey’s multiple comparisons test (α = 0.05) for a comparison of more than two groups. Error bars indicate the standard deviation (SD). Significance is indicated as follows: *p < 0.05; **p < 0.01; ***p < 0.001; ****p < 0.0001.

Number of CpG-sites, length, GC-content, CpG-density, and the promoter activity of the UCOE elements were correlated with the anti-silencing activity by using linear regression analyses in Graphpad Prism. The anti-silencing activity was defined as the relative percentage of eGFP-expressing cells (with day 0 accounting for 100% activity) of cells transduced with SFFV-based lentiviral vectors following undirected differentiation. The promoter activity of the CBX3 subfragments was defined as their ability to directly drive eGFP expression (measured as median fluorescence intensity (MFI)). The MFI was corrected for background eGFP expression of the negative control (G) by subtraction from each sample (i.e. G = 0) thereby yielding MFI_corr_. Subsequently, the MFI_corr_ was normalized to the vector copy number (MFI_corr_/VCN). For the correlation of promoter activity and anti-silencing activity, the data were adjusted to a linear scale by using the positive control (C-SG) as 100% and the negative control (SG) as 0%.

### Data availability

The datasets generated during and/or analysed during the current study are available from the corresponding author on reasonable request.

## Electronic supplementary material

Supplementary material
