# Supplementary material for: The CpG-sites of the CBX3 ubiquitous chromatin opening element are critical structural determinants for the anti-silencing function
Source: Sci Rep. 2017 Aug 11;7:7919. doi: 10.1038/s41598-017-04212-8 (PMC5554207; doi:10.1038/s41598-017-04212-8)
Supplement: Supplementary file 1 — Supplementary material [file 41598_2017_4212_MOESM1_ESM.pdf]

The CpG-sites of the CBX3 ubiquitous chromatin opening element are critical structural determinants for the anti-silencing function

Jessica Kunkiel [a,b], Natascha Gödecke [c], Mania Ackermann [b,d], Dirk Hoffmann [b], Axel Schambach [b,e], Nico Lachmann [b,d], Dagmar Wirth [b,c], Thomas Moritz [a,b,*]

[a] Reprogramming and Gene Therapy Group, REBIRTH Cluster of Excellence, Hannover Medical School, 30625 Hannover, Germany
[b] Institute of Experimental Hematology, Hannover Medical School, 30625 Hannover, Germany
[c] Model Systems for Infection and Immunity Group, Helmholtz Centre for Infection Research, 38124 Braunschweig, Germany
[d] Translational Hematology of Congenital Diseases Junior-Group, REBIRTH Cluster of Excellence, Hannover Medical School, 30625 Hannover, Germany
[e] Division of Hematology/Oncology, Boston Children's Hospital, Boston, MA, USA

***Corresponding author: Thomas Moritz**
E-mail: moritz.thomas@mh-hannover.de
Tel.: +49 511 532 5263
Fax: +49 511 532 5234

Supplementary Table S1: Quantitation of SG-based vector integrations.

Copy numbers (VCN)						
**Vector**	**mESC**			**miPSC**		
**SG**	3.87	3.31	3.04	0.69	0.32	0.96
**A2-SG**	4.13	3.16	2.98	0.28	2.79	0.65
**C-SG**	3.61	3.39	3.13	1.04	0.68	2.68
**C(1-339)-SG**	2.50	2.80	2.10	0.54	0.78	0.68
**C(1-508)-SG**	2.01	2.29	2.43	1.05	0.60	0.67
**C(85-508)-SG**	2.20	2.33	3.08	2.05	1.31	0.79
**C(170-508)-SG**	2.69	2.33	1.76	1.5	0.57	1.21
**C(340-508)-SG**	1.86	2.15	2.22	0.93	0.60	0.93
**C(503-679)-SG**	2.48	2.42	2.67	1.44	0.74	2.35

* Data represent three independent transductions

Supplementary Fig. S1: Activity of the CBX3 subfragments in pluripotent stem cells.

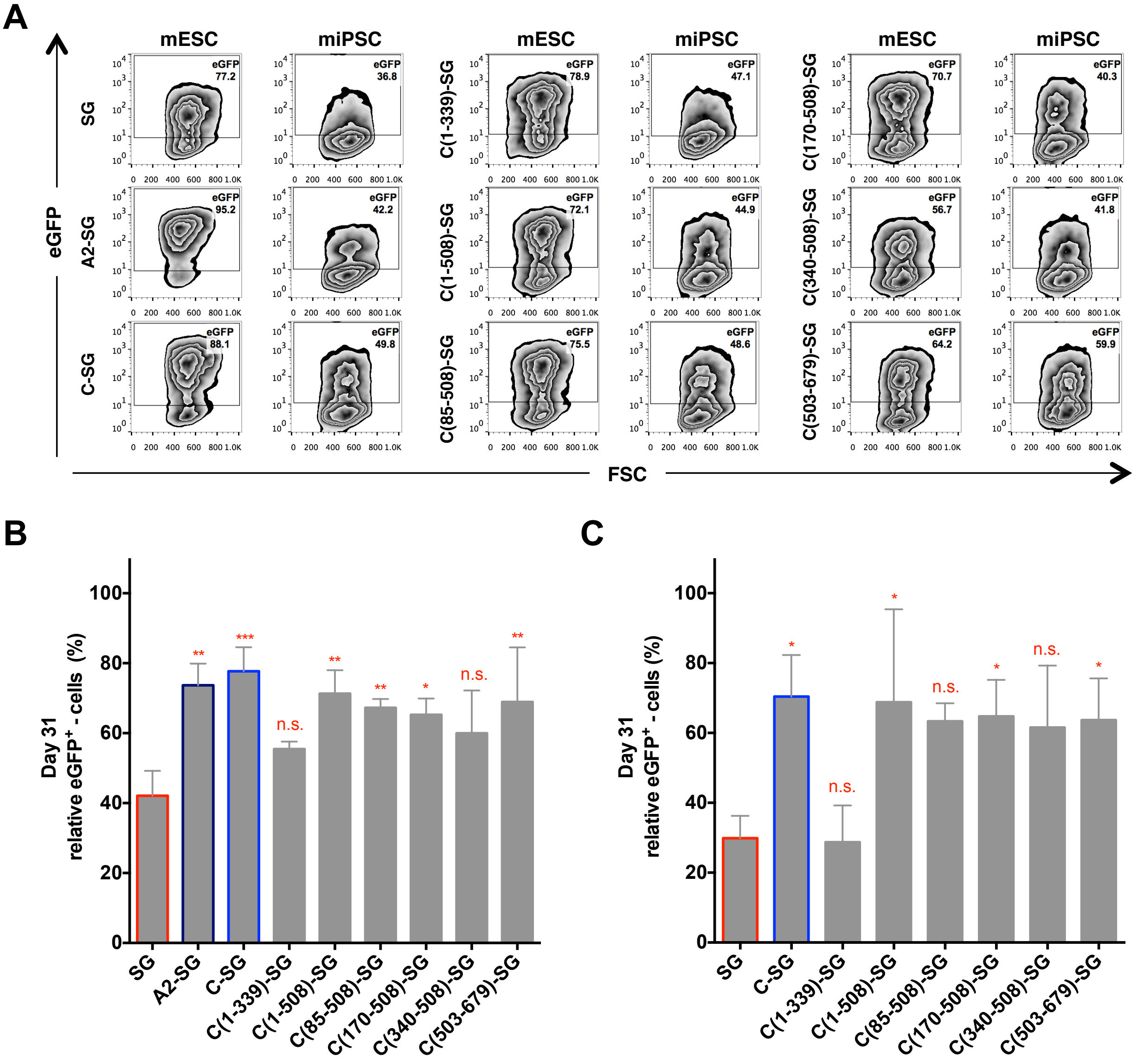

(**A**) Representative flow cytometric analysis of eGFP expression in SSEA-1-positive mESCs and miPSCs at day 3 after transduction. (**B**) Relative percentage of eGFP-positive cells on day 31 after transduction in mESCs and (**C**) miPSCs. Activity of the elements was compared to the SG negative control. Data represent independent experiments n = 3; mean ± SD; * $p < 0.05$, ** $p < 0.01$, *** $p < 0.001$ as determined by one-way ANOVA.

Supplementary Fig. S2: Alignment of synthetic scrambled CBX3 elements

CBX3	1	CCCGGGAGGTGGTCCCTGCAGT TACG CCA A TGAT AACCCCCGCCAGAAAA A TCTTAG TAGCCTT CCCTT TT TGT
CBX3scrCpG	1	GACGG AACCT AGTA TTC TCCT ACTCGC ACCCT AG TGGGCCCGGCTGTTGGCCCTTCT TAGCCCTCT CAGGCTGG
CBX3scr	1	GGGGGGCCCTCCCTGGGGAGCCCAGGGGCCCCCT GAAGGGGGCCCTAGCTTGCCAGCTAAACT GAAGGCCAGGC
CBX3	75	T TTCCGTGCCCC AACT CGGCGGAT TGAC TCGGCCCC TTCCGGAAACACCCGAAT CAACT TCT AGTCA AA TT A TT
CBX3scrCpG	75	CCCCCGTA GCCCTGGGAGGCA ACAGGGT CCGAGCCT CCTCGGCCCTGATCGGCTCTGGGCCTCCCTGC AGCAGA
CBX3scr	75	CCTGGGGCCCCCCTGT CCCTG CACACT GGGGGCCCCCTTGC TA CACTCAGCACTCACCAGGCCT TAGGGGGCCC
CBX3	149	GT TCACGCCGCAATGA CCCACCCCTGGCCCGCGTCTGTGGAACT GAC CCCTGGTGTACAGG AGAGTTCG CTGC
CBX3scrCpG	149	GCTCCCGT CGCACTCCTGCCTG AACCTGCCGCGCT AAGTGGCAGTCC T CAGGCTTCCTTG TCAGCCACGCCAC
CBX3scr	149	TG TAGGCT GCCTCTGGTGGCCCCTTGGCT TGCCCTGCCAAGCA GGGGGGCCACCTTGCCCCTGGGCCCCCCCC
CBX3	222	TGA AAGTGGT CCCAA AGGGGT AC TAGT TT TTA AGCTCCCA ACTCCCCCTCCCCCAGCGTCTGGAGGAT TCCACA
CBX3scrCpG	222	TGGCCTCCC AGCT CAGT AGT AGAAGT CCAGCCCCTTA T TGTCCC TAGTCCCTA TC TCGCAATCCA ATT GCCCTT
CBX3scr	222	AGGGTGGGGGGCCCTCT TT CTGAA GCCAGT AGCTCTA GCAGCTGCT TCCCTTGGGCAGTCCCTAA CTCCCACCT
CBX3	296	CCCTCGCACCG CAGGGGCGAGGAAGTGGGCGGAGT CCGGTTT TGGCGCCAGCCGC TGAGGCTGCCAAGCAG
CBX3scrCpG	296	TGGCCGCCCCGGCCCA A CGAACCCACT GACGGGGCCCGA TCAGGCCGCTGCT CGCA AGCAGTCT TGTGGCC
CBX3scr	296	TC AGCAGTGGCCAGTG GGCCCCTGGCCCCAGC TGT GCCACAGCA GCCTGGTGCC AGCTGC TCCCCACCCTT
CBX3	367	AA AAGCCACCGCTGAGGAGACTCCGGTCAC TGTCCTCGCCCCGCCT CCCCCT TCC CTCCCC TTGGGGACCACCG
CBX3scrCpG	367	T AGAGTT GTCGTCTGCAAGCTCT CGGACAGGCACCTCGGGACGAGCCA AT ACT AC CCTGGAGCCAGCCCAGCCG
CBX3scr	367	AC TGCACCAAGACTT ACT ACTCC TA CGCAGAGCCTGTCT TGCA AGGAGGAGGGGGCCAGGCTGCAGCTCAGTGG
CBX3	441	GGCGCCACGCCGCGAACG TTAAGTGCCGCGGTCGTCGGCGCCTCCGCCCTC CCCCTT GGGCCCCAAT T CCCAG
CBX3scrCpG	441	A TCGGTCCGCCCGGCCACGGCAGCTGTCGCGATCGCCGCCGGCACCGAA TCT AGGT AGAGGCCAGAGGGGGCCC
CBX3scr	441	C TA AGGCAGCCCACCAGGGCCCCCACCCT TTCCTCT CTGCGCAA TA CCCCCT ACTC TCCCA CT TAGGCT GGCC
CBX3	514	CGGGCGCGGCGCGCGGCCCCTCCCCCCGC CGGGCGCGCGCCCGCTGCCCCGCCCT TCGTGGCCGCCCGGCG T
CBX3scrCpG	514	CGA GCGCGACGCGCGGCCCTCCAGT CCGGCGT CCGCGCGGACGGCTGCCCGGGCACCGTT CTCG ACCGCCGG
CBX3scr	514	A AGGT GGACT GCAGCCTGGGCCCACT GCCAGC AGCCTT GCCT T TCCTGT AGGCCACCT AGCCCAGGGCC CTT
CBX3	586	GGGCGGTGC CACCCC TCCCCCCGGCGGCCCCGCGCGCAGCT CCCGGCTCCCTCCCCCTTCGGAT GTGGCTTGA
CBX3scrCpG	586	GCT CGT AGGGCCT CCCCT TCTCGGCGGCC TCGCGCGT TT ACACCGGGTAGCCACCCCTCCGT TTGGCCAAGCT
CBX3scr	586	T GGCA AGCCC CTGGGGCCCCTGGCAA TA GCCT TC CTGGTGGCCCCCTTGCTCCA CCACAAGTGCT CAGACTG
CBX3	659	GCTGT A GGCGCGG AGGGCCGG
CBX3scrCpG	659	GCCAC CCT CGCGA GCC TCCGC
CBX3scr	659	GGGGCCCC CCC AGGGC CTCCC

The sequence of the original CBX3 is shown in alignment with the two scrambled versions of the CBX3. The CBX3scrCpG element was obtained by shuffling the sequence at random whilst keeping the CpG-sites at their original position (highlighted parts). Random shuffling of the complete DNA sequence (CpG-sites destroyed) gave rise to the CBX3scr element.

Supplementary Table S2: Quantitation of CBX3scr(CpG)-containing SG-based vector integrations.

Copy numbers (VCN)						
**Vector**	**mESC**			**miPSC**		
**G**	2.69	2.48	1.91	0.61	0.87	1.13
**C-SG**	2.35	2.15	2.26	0.36	0.60	0.75
**CscrCpG-SG**	2.19	2.14	2.16	0.52	0.76	0.60
**Cscr-SG**	2.60	2.32	2.71	0.79	0.91	0.66

* Data represent three independent transductions

Supplementary Fig. S3: Activity of the synthetic scrambled CBX3 elements in pluripotent cells.

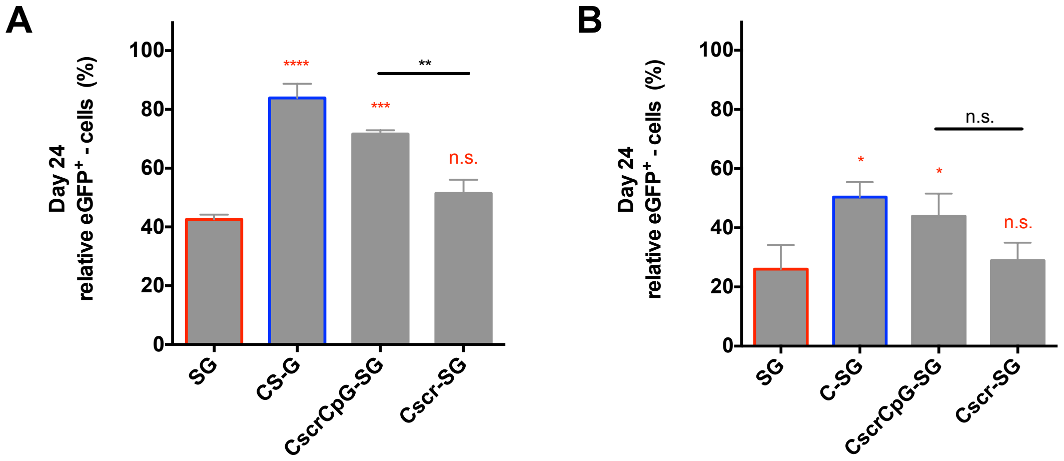

The relative percentage of eGFP-expressing cells was determined by flow cytometry on day 24 in (**A**) mESCs and (**B**) miPSCs. Activity of the elements was compared to the SG negative control. Data represent independent experiments n = 3; mean ± SD; * $p < 0.05$, ** $p < 0.01$, *** $p < 0.001$, **** $p < 0.0001$ as determined by one-way ANOVA.

Supplementary Table S3: Expression intensity of synthetic scrambled CBX3 elements in pluripotent stem cells.

	Median fluorescence intensity (MFI)			
	mESC		miPSC	
**Vector**	**d3**	**d24**	**d3**	**d24**
**SG**	46±6	39±8	34±4	32±6
**C-SG**	164±45***	153±35**	66±7***	53±4***
**CscrCpG-SG**	31±0.5	31±0.9	26±1	24±3
**Cscr-SG**	47±2	29±2	32±4	33±3

Activity of the subfragments was compared to the SG negative control. Data represent independent experiments n = 3; mean ± SD; * $p < 0.05$, ** $p < 0.01$, *** $p < 0.001$, **** $p < 0.0001$ as determined by one-way ANOVA.

Supplementary Table S4: Quantitation of eGFP-only based vector integrations.

Copy numbers (VCN)						
**Vector**	**mESC**			**miPSC**		
**G**	5.02	3.17	3.72	0.47	0.75	0.63
**C-G**	5.89	5.57	2.99	0.33	0.83	1.22
**C(1-339)-G**	5.89	3.70	3.29	0.55	1.07	1.00
**C(1-508)-G**	9.42	7.49	8.60	2.06	2.70	2.06
**C(85-508)-G**	9.99	7.13	6.07	0.88	1.84	1.37
**C(170-508)-G**	7.95	4.92	4.89	0.7	1.11	1.18
**C(340-508)-G**	12,26	7.15	7.99	1.7	2.26	2.76
**C(503-679)-G**	4.06	5.50	5.19	0.84	2.46	1.32
**CscrCpG-G**	5.93	5.36	6.30	1.97	1.69	3.11

* Data represent three independent transductions

Supplementary Fig. S4: Promoter activity of the CBX3 elements.

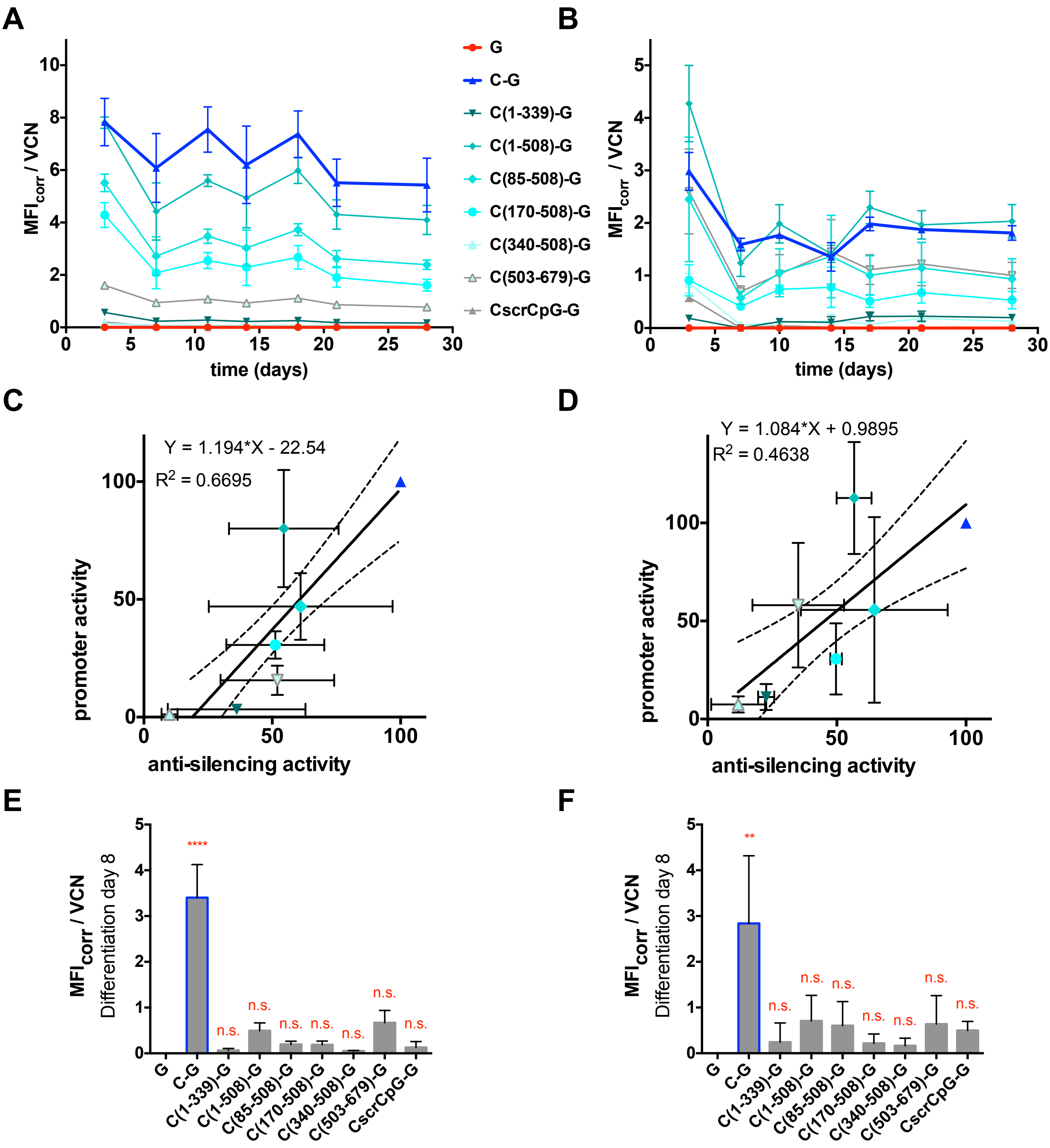

For 28 days the median fluorescence intensity (MFI) was monitored by flow cytometry, corrected for background eGFP (G=0; MFI_{corr}) and determined per vector copy number (VCN; MFI_{corr}/VCN) in (**A**) mESCs and (**B**) miPSCs. (**C-D**) The activity of the CBX3 subfragments in differentiated cells transduced with SG-based vectors (anti-silencing activity) was correlated with their ability to directly drive eGFP expression in pluripotent cells (promoter activity; day 28) with activity adjusted to a linear scale using SG = 0% and C-SG = 100% for (**C**) mESCs and (**D**) miPSCs. (**E-F**) After 8 days of undirected EB-based differentiation the MFI_{corr} per VCN of eGFP-expressing cells was monitored in SSEA-1 negative cells on day 8 for (**E**) mESCs and (**F**) miPSCs. Activity of the elements was compared to the SG negative control. Data represent independent experiments n = 3 (n = 2 for CBXscrCpG-G in F); mean ± SD; ** $p < 0.01$, **** $p < 0.0001$ as determined by one-way ANOVA.

Supplementary Fig. S5: Activity of the CBX3 element at two defined chromosomal sites in pluripotent stem cells.

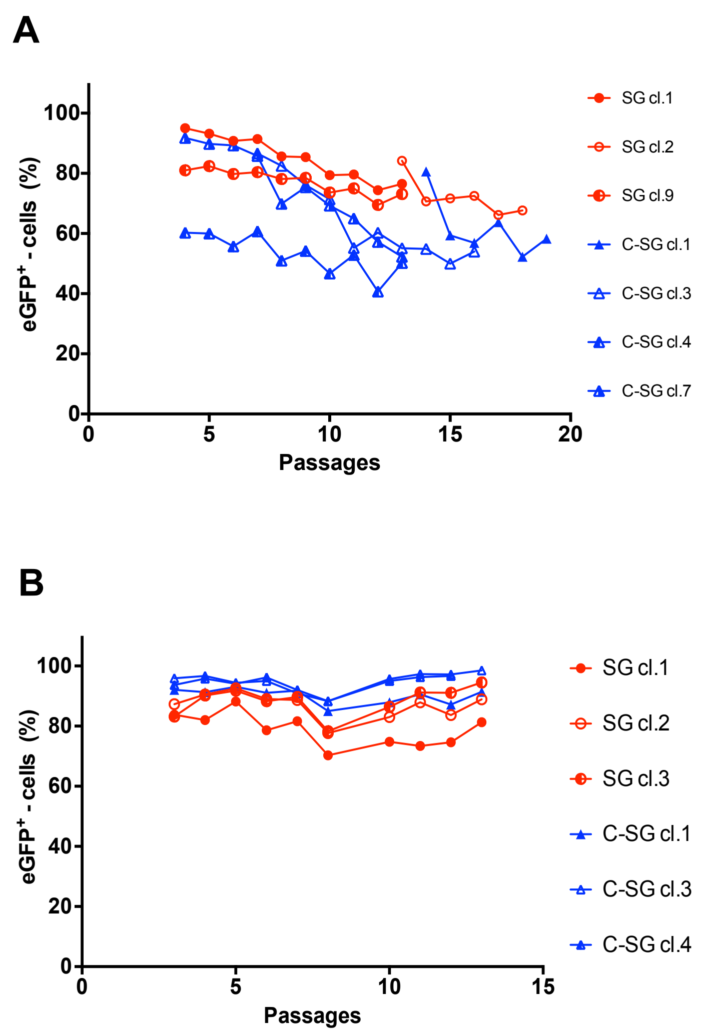

(**A**) The ROSA26 locus was targeted in mESCs with either SFFV.eGFP (SG) or CBX3.SFFV.eGFP (CSG) and the eGFP expression was determined by flow cytometry for 10 (or 6 for SG cl. 2 and CSG cl.1) successive passages in 3 (SG) and 4 (CSG) cell clones. (**B**) eGFP expression was also determined by flow cytometry in TIGRE-targeted mESCs (SG: 3 clones and CSG: 3 clones) for 10 passages.

Supplementary Table S5: **Primer sequences and combinations.**

Primer	Restriction site	Sequence
CBX3 1 fwd	CTCGAG	CCCGGGAGGTGGTCCCTGC
CBX3 2 fwd	CTCGAG	CCAACTCGGCGGATTGACTC
CBX3 3 fwd	CTCGAG	CCCTGGCCCGCGTCTGTGG
CBX3 4 fwd	CTCGAG	GCGCCAGCCGCTGAGGCTG
CBX3 5 rev	GCTAGC	CAAAACCGGACTCCGCCCA
CBX3 6 rev	GCTAGC	AATTGGGGCCCAAGGGG

Subfragment	Primer combination
CBX3(1-339)	1 fwd + 5 rev
CBX3(1-508)	1 fwd + 6 rev
CBX3(85-508)	2 fwd + 6 rev
CBX3(170-508)	3 fwd + 6 rev
CBX3(340-508)	4 fwd + 6 rev
